# A field efficacy and safety trial in the Netherlands in pigs vaccinated at 3 weeks of age with a ready-to-use porcine circovirus type 2 and *Mycoplasma hyopneumoniae* combined vaccine

**DOI:** 10.1186/s40813-017-0070-5

**Published:** 2017-11-09

**Authors:** Luuk Kaalberg, Victor Geurts, Rika Jolie

**Affiliations:** 1 De Graafschapdierenartsen bv, Schimmeldijk 1 Vorden, 7251 MX Vorden, The Netherlands; 2 MSD Animal Health, Wim de Korverstraat 35, 5831 AN Boxmeer, The Netherlands; 3 Merck Animal Health, 2 Giralda Farms, Madison, NJ 07940 USA

**Keywords:** PCV2, *Mycoplasma hyopneumoniae*, Bivalent vaccine, Randomised controlled field trial

## Abstract

**Background:**

Respiratory diseases impair the health and welfare of growing pigs and impacts farmers’ gains worldwide. Their control through a preventative medical approach has to be tailored according to the pathogens identified at farm level. In the Netherlands, several studies have emphasized the prominent role of *Mycoplasma hyopneumoniae*, Porcine Circovirus type 2 and Porcine Reproductive and Respiratory Syndrome Virus in such respiratory conditions. Further to the arrival on the Dutch market of the first commercially available bivalent vaccine against PCV2 and *M. hyopneumoniae*, Porcilis® PCV M. Hyo, a trial was designed to evaluate its safety and efficacy under local field conditions.

**Material and methods:**

In a conventional farrow-to-finish 170-sow farm with a history of respiratory diseases and demonstrated circulation of both *M. hyopneumoniae* and PCV2, 812 piglets were randomised and included at weaning in either of the three following groups: PCVM (vaccinated with Porcilis® PCV M. Hyo), FLEX (vaccinated with CircoFLEX® and MycoFLEX®) or NC (negative control, injected with placebo). Piglets were vaccinated at 3 weeks of age (day 0) and a subset was bled and weighed at regular intervals up to slaughter. Lung slaughter checks were only performed on 64% of the pigs included on day 0.

**Results and implication:**

No side effect of injection was observed in any of the three groups. Average daily weight gain was improved in both vaccinated groups as compared to the NC group, over the finishing period as well as from wean-to-finish. The PCVM group had a significantly lower PCV2 viremia area under the curve than the two other groups, and a significant reduction in the severity of the pneumonia-like lesions was observed at slaughter in the pigs of the PCVM group. A conservative estimate of the economic benefit of that vaccine was 2.84 € per finisher. This trial confirms that the vaccine is efficacious against the health and growth effects of PCV2 and *M. hyopneumoniae*, of practical advantage (single injection of a bivalent product) and well tolerated.

## Background and regional context

Porcine Circovirus type 2 (PCV2) and *Mycoplasma hyopneumoniae* are two of the dominating pathogens in the global industry of fattening pigs. PCV2 infection can produce overt clinical diseases as well as subclinical growth retardation, the latter being considered as the most common form of PCV2 infection worldwide [1]. *M. hyopneumoniae* induces a chronic lung infection which impacts growth [2]. Both pathogens have developed immune evasion strategies that are still only partially understood [3, 4]. In growing pigs, these two pathogens, through their ability to interfere with effectors of both the innate [5, 6] and adaptive immune response [5, 7], tend to foster infections with other pathogens. Being both slow growers [5, 8] and immunity-wise discrete invaders [5, 9], PCV2 and *M. hyopneumoniae* have proven difficult to control by vaccination so far [10, 11].

In Europe, extensive epidemiological studies have been published on infectious risk factors of respiratory diseases. In France, it was shown that PCV2 seems to be of lesser importance in these disorders than *Mycoplasma hyopneumoniae* [12]. In Belgium, a recent cross-sectional study explored several infectious factors potentially associated with lung lesions at slaughter, but failed to include PCV2 among them, showing that it was not considered a relevant respiratory pathogen [13]. In the Netherlands on the other hand, PCV2, *Pasteurella multocida*, *Mycoplasma hyopneumoniae*, and swine influenza viruses (SIVs) were all found in a significantly higher frequency in herds having an elevated proportion of lung lesions (pneumonia) at slaughter, but with no clinical signs of post-weaning multisystemic wasting syndrome (PMWS) [14]. More recently, two reports added to this knowledge: i. in 27 Dutch farms with respiratory clinical signs, between November 2013 and October 2015, oral fluid sampling allowed to observe that finishers (pigs of 19–24 weeks of age) were positive for the genome of *M. hyopneumoniae* (> 75% of farms), PRRSV (35%), PCV2 and *Actinobacillus pleuropneumoniae* (100%)^1^; ii. in respiratory diseases outbreaks on 412 Benelux farms over a 4-year period, diagnostic test results confirmed the dominance of *M. hyopneumoniae*, PRRSV and PCV2 in fattening pigs, without seasonal variation.^2^ Another study conducted in Germany also evidenced that PCV2 is a major component of respiratory diseases in that region [15]. However, the role of PCV2 in pneumonic lesions is rarely evoked in the field, where lung slaughter checks are exclusively considered as a monitoring tool to assess the prevalence of enzootic pneumonia; of note is that all lung lesion scoring systems are positively and significantly correlated [16]. Also, respiratory pathogens have been shown to interact with each other [17], e.g. *M. hyopneumoniae* as well as PRRSV have been shown to potentiate PCV2-associated lesions. One may also interfere with vaccination against the other. Under experimental conditions, in pigs dually infected with *M. hyopneumoniae* and PCV2, the single vaccination against either pathogen has not been found to have an impact on the lung lesions induced by the other one. Conversely, piglet vaccination against *M. hyopneumoniae* was found to reduce PRRSV replication in co-infected piglets (as compared to non-vaccinated ones) (see [18] for a review). More lately, bivalent and combined vaccines against PCV2 and *M. hyopneumoniae* have become commercially available. While the practical interest of such products is obvious in terms of labour (a single injection as opposed to 2 injections for the monovalent vaccines), their preventative efficacy is regularly questioned in the field — partly because the epidemiology of respiratory diseases may vary locally. Comparative field data on the two single-shot 2-valence vaccines are scarcely available to the European prescribers. The present study’s aim is providing such data, particularly adding to the European Medicines Agency’s public assessment report on Porcilis® PCV M. Hyo, the first ready-to-use bivalent vaccine against PCV2 and *Mycoplasma hyopneumoniae* 3.

## Case farm and trial setting

In the fall of 2014, a conventional 170-sow Dutch farrow-to-finish swine farm managed in a 2-week batch system with a history of PCV2-subclinical infection and of enzootic pneumonia was selected for a field trial. The former condition was characterized by a decreased average daily weight gain (ADWG) in the finishing phase over year 2014, as defined by [1]. Also, pre-trial investigations identified a PCV2 serological profile presenting an increase in antibody titers starting around 10 weeks of age; 16–22 week old pigs were also PCV2 PCR-positive in oral fluids, showing together an early and prolonged PCV2 circulation in the herd. The *M. hyopneumoniae* condition was evidenced by an elevated lung lesion score (LLS) assessed at slaughter (Madec scoring system [19]), which was above the average LLS of the slaughterhouse. Also, *M. hyopneumoniae* serology was partially positive in 16 week-old pigs, and completely positive in 22 week-old pigs; furthermore, the saliva samples of 16 week-old finishers were PCR-positive for *M. hyopneumoniae*. These results demonstrate that a herd infection took place during finishing. On that farm, sows, nursery and finisher pigs were housed in separate stables, although in a single site.

On the day of inclusion in the trial (d0), piglets of on average 21 days of age (16–26 days) were individually weighed and identified with their unique numbered ear tags (piglets appearing unhealthy were not identified, hence discarded). Within each litter, piglets were assigned to either of the three study groups.

Group 1 (PCVM, *n* = 269): vaccination with Porcilis® PCV M Hyo (MSD Animal Health, Boxmeer, the Netherlands), according to the product SPC^4^: 2 ml via the intramuscular (IM) route, in the neck.

Group 2 (FLEX, *n* = 267): vaccination with a single injection of CircoFLEX® and MycoFLEX® (Boehringer Ingelheim, Ingelheim, Germany), according to the product SPC^5^: 2 ml (2 × 1 ml dose) via the IM route, in the neck.

Group 3 (NC, *n* = 276): injection of saline, 2 ml via the IM route in the neck. Group 3 is the negative control group.

Because of the farm’s 2-week batch production management, 6 batches of piglets were included in the trial, between Dec. 18, 2014 and March 5, 2015 (72 litters and 812 piglets in total). In half of these batches (#1, #3 and #5), around 10 piglets from each treatment group were randomly selected to be blood sampled on regular intervals (at 3, 10, 18 and 22 weeks of age). All trial piglets were also individually weighed on these dates. On d0, piglets selected for blood sampling received a red-paint dot on their ear-tag; blood sampling was performed after group allocation but before vaccination. There was no statistically significant difference between groups for age, and weight at inclusion, and sex (see Table 1).

**Table 1:** Overview of the number of animals, age at inclusion, sex and weight at vaccination or injection, per treatment group

	Control (*N* = 276)	FLEX (*N* = 267)	PCVM (*N* = 269)	Overall (*N* = 812)
Age (days)				
16	3 (1.1%)	3 (1.1%)	2 (0.7%)	8 (1.0%)
17	39 (14.1%)	35 (13.1%)	40 (14.9%)	114 (14.0%)
18	44 (15.9%)	42 (15.7%)	43 (16.0%)	129 (15.9%)
19	28 (10.1%)	27 (10.1%)	28 (10.4%)	83 (10.2%)
20	3 (1.1%)	3 (1.1%)	3 (1.1%)	9 (1.1%)
21	15 (5.4%)	16 (6.0%)	14 (5.2%)	45 (5.5%)
22	26 (9.4%)	25 (9.4%)	27 (10.0%)	78 (9.6%)
23	61 (22.1%)	58 (21.7%)	60 (22.3%)	179 (22.0%)
24	32 (11.6%)	33 (12.4%)	27 (10.0%)	92 (11.3%)
25	22 (8.0%)	22 (8.2%)	22 (8.2%)	66 (8.1%)
26	3 (1.1%)	3 (1.1%)	3 (1.1%)	9 (1.1%)
Gender				
Female	144 (52.2%)	136 (50.9%)	122 (45.4%)	402 (49.5%)
Male	132 (47.8%)	131 (49.1%)	147 (54.6%)	410 (50.5%)
Weight (kg)				
N	276	267	269	812
Mean	5.6	5.6	5.7	5.6
Standard deviation	1.54	1.56	1.57	1.56
Minimum	1.6	1.7	1.9	1.6
Maximum	10.2	10.2	10.0	10.2

Pigs from different groups were commingled throughout the study, according to the current farm practices.

## Assessment of vaccine safety and efficacy

Although safety was not the primary objective for this study, the piglets were monitored for both local and systemic reactions immediately and 1 h after injections. No side effect was recorded in any of the three groups.

To assess the efficacy of the vaccines in the farms’ context, three primary parameters were used: PCV2 viremia (genomic serum load) for protection towards infection; lung lesion score at slaughter for prevalence and severity of enzootic pneumonia; and average daily weight gain (ADWG) in the finishing period for the control of both pathogens (PCV2 and *M. hyopneumoniae*). Mortality was recorded as a secondary parameter; this allowed, together with primary parameters and slaughterhouse information, to estimate the economic impact of the vaccines.

The PCV2 viral load was determined by the Boxmeer MSD R&D Service Laboratory by a PCV2-specific real-time polymerase chain reaction [20].

All sampled control animals became PCV2 viraemic during the study (see Table 2). In the FLEX group 67% became viraemic and 43% in the PCVM group. The PCV2 viraemia, expressed as area under the curve (AUC), in the PCVM group was significantly lower than in the control group (ANOVA: *p* < 0.0001) and in the FLEX group (ANOVA: *p* = 0.0022). The FLEX group also had a statistically significant (*p* < 0.0001) lower viraemia compared with the control group. Two pigs (both from the FLEX group) were viraemic at vaccination and were not included in this analysis.

**Table 2:** Mean PCV2 viraemia (expressed in log10 copies/μl DNA extract) by sampling time after admission and AUC, by trial group

	PCVM group	FLEX group	Negative control group
Admission	0.00 ± 0.00	0.00 ± 0.00	0.00 ± 0.00
Week 7	0.21 ± 0.52	0.75 ± 1.25	2.62 ± 2.02
Week 15	0.23 ± 0.69	0.92 ± 1.31	1.93 ± 1.12
Week 19	0.04 ± 0.12	0.29 ± 0.75	1.38 ± 1.29
AUC^a^	2.99/ 0.00^a^	12.11/ 3.90^b^	33.99/ 36.64^c^

Values with different superscript letters within the same row are statistically significantly different (ANOVA, ^ab^ *p* = 0.0022; ^ac^ *p* < 0.0001; ^bc^ *p* < 0.0001)

^a^Mean/Median

During the trial, wasting piglets were observed on the farm (contemporary batches), suggesting that PCV2 viral pressure was still present, although limited (overall mortality was low).

Lung lesion score was evaluated according to the Madec system, and a mixed ANOVA model was used to analyze these scores. Equivalent Goodwin scores [21] were calculated from the Madec scores and statistical analysis was performed on the log10-transformed total lung lesion scores^6^. The sample size of 300 pigs per group had a power above 80% to detect a difference of 0.15 in log10-transformed LLS (Goodwin system). This size was not achieved, since lungs from only 64% of pigs included on d0 were checked (*n* = 518); the rest of them were not scored for several reasons (death, loss of the ear tag or missed in the slaughter line). LLS can be interpreted however, since the proportion of pigs lost over the trial duration was comparable in each group (96 [36%] in the PCVM group, 104 [39%] in the FLEX group and 94 [34%] in the NC group), mortality rate among each group was not statistically different: 36 pigs died or were culled during the study (3.3% (9/269) in the PCVM group, 4.5% (12/267) in the FLEX group and 5.4% (15/276) in the NC group) and the numerical difference in total LLS (Goodwin) between groups exceeds 0.15. There were differences in the proportion of lungs with enzootic pneumonia-like lung lesions between groups, with a proportion that was nearly double in the NC group compared with both vaccinated group (see Table 3). Also, average total LLS was higher in the NC group (0.4 ± 1.4) compared to both vaccinated groups (PCVM: 0.1 ± 0.6; FLEX: 0.2 ± 1.0). The difference between both vaccinated groups was not significant (mixed model ANOVA, *p* = 0.4781), but the difference in total LLS was significant between the PCVM and the NC groups (*p* = 0.0163). Finally, the severity of these lesions was significantly lower in the PCVM group as compared to the NC group (NC-PCVM = 0.14; [95%CI 0.03–0.26], *p* = 0.0157). This difference was not significant when the FLEX and NC groups were compared (NC-FLEX = 0.11; [95% CI: 0.001–0.23]; *p* = 0.0528), nor when PCVM and FLEX groups were compared (FLEX-PCVM = 0.03; [95% CI: 0.09–0.14]; *p* = 0.6507).

**Table 3:** Distribution of the lung lesion scores by treatment (n [%]), according to the Madec scoring system and the extrapolation to the Goodwin scoring system

		PCVM group (*N* = 173)	FLEX group (*N* = 163)	NC group (*N* = 182)
Madec scoring system (max. 28)	Missing^a^	20 [11.6]	18 [11.0]	28 [15.4]
	Score 0	142 [82.1]	133 [81.6]	131 [72.0]
	Score 1–3	10 [5.8]	9 [5.5]	17 [9.3]
	Score 4–6	1 [0.6]	2 [1.2]	4 [2.2]
	Score 7–9	0 [0.0]	0 [0.0]	1 [0.5]
	Score 8–12	0 [0.0]	1 [0.6]	1 [0.5]
	Maximum score	5	10	11
	Proportion of lungs with EP-like lesions	6.3%	7.4%	12.6%
Goodwin scoring system (max. 55)	Score 0	142 [82.1]	133 [81.6]	131 [72.0]
	0 < Score ≤ 5	8 [4.6]	9 [5.5]	15 [8.2]
	5 < Score ≤ 10	2 [1.2]	2 [1.2]	5 [2.7]
	Score > 10	1 [0.6]	1 [0.6]	3 [1.6]
	Maximum score	12.5	21.25	25

^a^Missing animals were those that had been slaughtered before the lung scoring team arrived

*M. hyopneumoniae* serology, performed with the Swine HerdChek® M. hyo IDEXX kit, confirmed that *M. hyopneumoniae* exposure occurred during the finishing period, with a limited challenge (3% of positive pigs in the NC group on week 22). The fact that the reduction in the severity of EP-like lesions was only significant in the group vaccinated with Porcilis® PCV M Hyo might have an economic significance, considering that a significant (*p* < 0.001) negative correlation has been demonstrated between pneumonia score and growth, with an ADWG loss of about 0.7% for each point of pneumonia increase [22].

For the analysis of the Average Daily Weight Gain (ADWG), a sample size of 300 in each treatment group had 80% power to detect a difference in means of 25 g/d between vaccinated and control groups. This size was not achieved at inclusion, but the measured ADWG over the finishing period exceeded this value (see Table 4). Hence, a mixed ANOVA model^7^ was used for statistical analysis. All animals were weighed individually with a calibrated scale. The weighing prior to slaughter was done on the same day for all animals of the same farrowing batch, one or a few days before the first pigs of that same batch were shipped to slaughter.

**Table 4:** Average Daily Weight gain improvement/reduction between treatment groups over the finishing period and overall (* *p* = 0.0004 **p = 0.0014)

Compared groups	ADWG over the finishing period (g/d)	Reduction of ADWG over the finishing period	Wean-to-finish ADWG (g/d)	Reduction of ADWG from wean-to-finish
PCVM vs. NC	PCVM: 842	−38 g/day* and 95% CI [−57, −19]	PCVM: 660	−24 g/day** and 95% CI [−37, −11]
	NC: 803		NC: 634	
FLEX vs. NC	FLEX: 827	−26 g/day* and 95% CI [−44, −7]	FLEX: 650	−17 g/day** and 95% CI [−30, −3]
	NC: 803		NC: 634	
PCVM vs. FLEX	PCVM: 842	−12 g/day and 95% CI [−31, 7]	PCVM: 660	−8 g/day and 95% CI [−21, 6]
	FLEX: 827		FLEX: 650	

There was no statistically significant difference in the ADWG over the nursery period between groups (data not shown), confirming that neither vaccine has a detectable negative impact on growth performance shortly after injection. Over the finishing period (10–22 weeks of age), there was a highly significant difference between both vaccinated groups and the NC group (*p* = 0.0004), but not between the PCVM and FLEX groups (see Table 4). Overall (weeks 3–22), the difference between both vaccinated groups and the NC group was also highly significant (*p* = 0.0014). Although not statistically significant, both during finishing and overall, ADWG was numerically higher in the PCVM group than in the FLEX group. ADWG, a primary efficacy parameter for the prevention against PCV2 and *M. hyopneumoniae* infections, was significantly higher in the finishing period for both vaccinated groups, compared to the control group, which confirms the interest of early vaccination of piglets with a bivalent vaccine on a farm where both pathogens are circulating.

The estimation of the improvement of the farmer’s income was based on the results in the PCVM group (ADWG and mortality) and the average Dutch economical production figures, compiled by the Wageningen Economic Research institute: the 38 g/d improvement of the ADWG corresponds to €1.37 more income per finisher; the 2-point lower mortality rate produces a €1.47 gain. In total, the trial at least allowed to estimate an income improvement of € 2.84 per finisher.^8^ This is probably a conservative estimate, since individual feed intake could not be measured and potential gains in food conversion rate (FCR) are not included in the present economical calculation.

## Conclusion

On a conventional Dutch farrow-to-finish farm with good management and a good health status, where PCV2 and *M. hyopneumoniae* were diagnosed as the prominent infectious risk factors for respiratory disease, a randomised field trial was performed. Three treatments were compared: piglets vaccinated at 3 weeks of age with Porcilis® PCV M Hyo bivalent ready-to-use vaccine, or with the FLEX combination vaccine (doses of the CircoFLEX® and MycoFLEX® vaccines mixed into a single injection), or with a placebo injection. Both vaccines significantly reduced the negative effect of both pathogens on growth performance, as measured by the ADWG over the finishing period, and overall. A significant reduction in the severity of the pneumonia-like lesions was only observed in the pigs vaccinated with Porcilis® PCV M Hyo. When viremia was expressed as AUC per group, the PCVM group had a significantly lower AUC value than both other groups. The FLEX group had a significantly lower AUC value than the NC group. This study further supports safety and efficacy data of Porcilis® PCV M Hyo bivalent ready-to-use vaccine.

## Abbreviations

ADWG: Average daily weight gain
FCR: Food conversion rate
LLS: Lung lesion score
PCR: Polymerase chain reaction
PCV2: Porcine circovirus type 2
PRRS: Porcine reproductive and respiratory syndrome
PRRSV: Porcine reproductive and respiratory syndrome virus
RTU: Ready-to-use
SIV: Swine influenza virus

## Footnotes

1 Van Dongen F. in Proceedings of the 24th International Pig Veterinary Society, Dublin Ireland, 7–10 June 2016, p. 287.

2 Vangroenweghe F. in Proceedings of the 24th International Pig Veterinary Society, Dublin Ireland, 7–10 June 2016, p. 151.

3 This EPAR is freely available online on the website of EMA: http://www.ema.europa.eu/ema/index.jsp?curl=pages/medicines/veterinary/medicines/003796/vet_med_000307.jsp&mid=WC0b01ac058008d7a8.

4 The summary of the European public assessment report of this vaccine is available online at http://www.ema.europa.eu/docs/en_GB/document_library/EPAR_-_Summary_for_the_public/veterinary/003796/WC500177275. pdf

5 The summary of the European public assessment report of this vaccine is available online at http://www.ema.europa.eu/docs/en_GB/document_library/EPAR_-_Product_Information/veterinary/000126/WC500062388.pdf

6 Both analyses included the sow as random effect and treatment as fixed effect.

7 The model considered the dam as a random effect and treatment, gender and their interaction as fixed effects. The initial weight was included in the model as a covariate.

8 Vaccination cost was not included in this calculation.
